# Identification and validation of a novel gene *ARVCF* associated with alcohol dependence among Chinese population

**DOI:** 10.1016/j.isci.2024.110976

**Published:** 2024-09-16

**Authors:** Xiaoqiang Shi, Yan Wang, Zhongli Yang, Wenji Yuan, Ming D. Li

**Affiliations:** 1 State Key Laboratory for Diagnosis and Treatment of Infectious Diseases, National Clinical Research Center for Infectious Diseases, National Medical Center for Infectious Diseases, Collaborative Innovation Center for Diagnosis and Treatment of Infectious Diseases, The First Affiliated Hospital, Zhejiang University School of Medicine, Hangzhou 310009, China; 2 Research Center for Air Pollution and Health, Zhejiang University, Hangzhou 310058, China

**Keywords:** Health sciences, Medicine, Medical specialty, Psychiatry, Natural sciences, Biological sciences, Neuroscience, Behavioral neuroscience

## Abstract

Alcohol dependence is a heritable disorder, yet its genetic basis and underlying mechanisms remain poorly understood, especially in Chinese population. In this study, we conducted gene-based and transcript-based association tests and found a significant association between *ARVCF* expression in the cortex and hippocampus of the brain and alcohol use in a cohort of 1,329 individuals with Chinese ancestry. Further analysis using the effective-median-based Mendelian randomization framework for inferring the causal genes (EMIC) revealed a causal relationship between *ARVCF* expression in the frontal cortex and alcohol use. Moreover, leveraging extensive European alcohol dependence data, our gene association tests and EMIC analysis showed that *ARVCF* expression in the nucleus accumbens was significantly associated with alcohol dependence. Finally, animal studies indicated that *Arvcf* knockout mice lacked conditioned place preference for alcohol. Together, our combined human genetic and animal studies indicate that *ARVCF* plays a crucial role in alcohol dependence.

## Highlights

• *ARVCF* was identified as a susceptibility gene for alcohol dependence
• *ARVCF* expression in the cortex and NAc causally affects alcohol dependence
• *Arvcf* is required for establishing alcohol dependence in mice

## Graphical abstract

**Figure:**
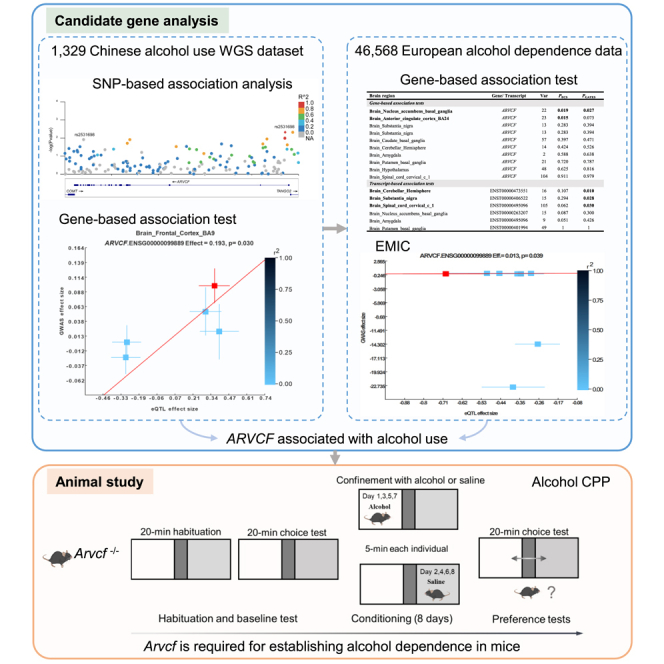

## Introduction

Excessive alcohol consumption is a leading risk factor for the global burden of disease, as it contributes significantly to morbidity and mortality. Moreover, alcohol use disorders (AUDs) are the most prevalent of all substance use disorders.^1^ It was estimated that alcohol use contributed to about 3 million deaths (5.3% of all deaths) and 132.6 million (5.1%) disability-adjusted life years worldwide in 2016.^2^ Alcohol dependence is responsible for continuing alcohol abuse-related behaviors, with approximately 12.5% of alcohol drinkers in the United States meeting the Diagnostic and Statistical Manual (DSM)-IV criteria for alcohol dependence in their lifetime.^3^ Meta-analysis of twin and adoption studies has revealed the role of genetic and environmental factors in alcohol dependence, with an estimated heritability of 50%.^4^ Despite the significant public health consequences, AUDs remain among the most undertreated mental disorders, partially due to the lack of effective treatment targets.^5^

Although a great number of genes and genetic variants are reported to be associated with alcohol use in European populations, relevant information in the Chinese population is still limited. Furthermore, only limited number of those identified susceptibility genes or variants were validated. Even alcohol is being consumed globally, but the majority studies on alcohol dependence and related clinical phenotypes such as alcohol consumption, AUD, or problematic alcohol use (PAU) are primarily focused on individuals of European ancestry and limited studies have been conducted on Asian subjects.^6^ For example, a recent meta-analysis analyzed a PAU phenotype by combining the phenotypes of AUD and alcohol use-related problems,^7^ which included 1,079,947 individuals, with nearly 1 million of European descent and only 13,551 of East Asian descent, indicating a substantial discrepancy in sample sizes. A similar pattern is observed for alcohol consumption phenotype. Presently, the largest genome-wide association study (GWAS) study on drinks per week included 3.4 million samples, with approximately 2.67 million of European descent and only 296,438 samples from East Asians.^8^ Studies focused on Chinese population are even scarcer,^9^^,^^10^ with only 533 male alcoholics in a recent alcohol dependence GWAS.^10^ In China, alcohol consumption is recently increasing faster than in other parts of the world,^11^^,^^12^ and this trend is projected to continue rising in the future.^13^ Recently, a larger-scale dataset for alcohol consumption from China Kadoorie Biobank became available, encompassing 168,050 individuals, but only two East Asian genetic variants (*ALDH2*-rs671 and *ADH1B*-rs1229984) were genotyped.^14^ Genetic architecture often varies between ancestries. According to a previous multi-ancestry study, polygenic risk scores developed in one ancestry, such as Europeans, performed poorly in other ancestral groups.^8^ Therefore, identifying novel genes that underlie alcohol dependence in non-European ancestries is important for gaining more insights and discovering new therapeutic targets.

Our previous research identified a novel functional variant, rs148582811, located in Armadillo Repeat gene deleted in Velo-Cardio-Facial syndrome (*ARVCF*), which regulates *ARVCF* expression and plays an important role in nicotine dependence.^15^ *ARVCF* is among the genes implicated in 22q11.2 syndrome, a neurogenetic disorder also known as velo-cardio-facial or DiGeorge syndrome. Individuals with chromosome 22q11.2 deletions are at an elevated risk of developing psychiatric conditions.^16^ The ARVCF protein belongs to the p120 catenin (p120ctn) subfamily, which includes four proteins encoded by four independent genes (p120ctn, encoded by *CTNND1*; δ-catenin, encoded by *CTNND2*; p0071, encoded by *PKP4*; and ARVCF, encoded by *ARVCF*). Previous studies have reported that p120ctn, δ-catenin, and p0071 play functional roles in dendritic and synaptic development.^17^^,^^18^ However, relatively few studies have focused on *ARVCF*. A recent study utilizing a primary rat hippocampal neuronal culture model indicated that increasing ARVCF-catenin levels resulted in increased dendritic length and branching,^18^ which was associated with alcohol addiction.^19^ To our knowledge, the potential role of the *ARVCF* gene in alcohol dependence has not been reported.

Here, we first conducted a gene-based association analysis and effective-median-based Mendelian randomization framework for inferring the causal genes (EMIC) analysis to determine if *ARVCF* is associated with alcohol use in a Chinese genome-wide sequencing dataset, and we then replicated the findings with an independent European alcohol dependence dataset. To further validate the role of the *ARVCF* gene in alcohol dependence, we then conducted an alcohol-induced conditioned place preference (CPP) test in CRISPR-Cas9-generated *Arvcf* knockout (KO) mice. Taken together, we first discovered that *ARVCF* is a susceptible gene for alcohol use, and then we confirmed that *ARVCF* plays an important role in alcohol dependence in mice.

## Results

### *ARVCF* variants associated with alcohol consumption in our Chinese cohort

Chinese participants included 834 individuals with alcohol drinking (mean age 40 ± 8 years) and 470 control individuals (mean age 42 ± 8 years) (Table S1). A total of 168 variants located in *ARVCF* (including ±2 kb flanking regions) were analyzed. After clumping the *ARVCF* locus for linkage disequilibrium (*r*^2^ < 0.01, within a 250 kb genomic window^20^), five single-nucleotide polymorphisms (SNPs) remained (Table S2). Our analysis indicated that the variant rs2531698 in *ARVCF* was significantly associated with drinks per week (β = 0.107, SE = 0.038, *p* = 4.79 × 10^−3^, Figure S1). Besides, another variant, rs116570619 in *ARVCF*, also showed an association with drinks per week (β = −0.409, SE = 0.163, *p* = 0.012, Figure S1). Notably, neither of these two variants has been reported in previous studies.

### Gene association analysis links *ARVCF* with alcohol use

We performed gene-based association analyses using the SNP-based association analysis results located in *ARVCF* from our Chinese samples and combined the Genotype-Tissue Expression (GTEx) brain expression quantitative trait loci (eQTL) data. The gene-based analysis revealed that *ARVCF* expression in the cervical spinal cord (*p*_GATES_ = 2.93 × 10^−4^, *P*_ECS_ = 0.013), frontal cortex (*p*_GATES_ = 0.004, *p*_ECS_ = 0.012), cortex (*p*_GATES_ = 0.007), and cerebellum (*p*_GATES_ = 0.029) was significantly associated with alcohol use (Tables 1 and S3). Additionally, transcript-based association analysis further implicated *ARVCF* expression in alcohol use, particularly in the hippocampus (*p*_GATES_ = 4.07 × 10^−4^, *p*_ECS_ = 0.022), cervical spinal cord (*p*_GATES_ = 6.40 × 10^−4^, *p*_ECS_ = 0.002), cortex (*p*_GATES_ = 0.022), and frontal cortex (*p*_GATES_ = 0.044) (Tables 1 and S4).

**Table 1:** Results of gene-based and transcript-based *ARVCF* association tests in Chinese samples

Phenotype	Brain region	Gene/transcript	Var	*p*_ECS_	*p*_GATES_
**Gene-based association tests**					

Drinks per week	Brain_Spinal_cord_cervical_c_1	*ARVCF*	45	0.013	2.93E-04
Alcohol drink	Brain_Frontal_Cortex_BA9	*ARVCF*	5	0.012	0.004
Alcohol drink	Brain_Cortex	*ARVCF*	31	0.082	0.007
Alcohol drink	Brain_Cerebellum	*ARVCF*	40	0.105	0.029

**Transcript-based association tests**					

Drinks per week	Brain_Hippocampus	ENST00000406522	13	0.022	4.07E-04
Drinks per week	Brain_Spinal_cord_cervical_c_1	ENST00000487793	25	0.002	6.40E-04
Alcohol drink	Brain_Cortex	ENST00000473551	8	0.055	0.022
Alcohol drink	Brain_Frontal_Cortex_BA9	ENST00000495096	41	0.054	0.044

Var, number of variants within the gene; *p*_ECS_, *p* value of ECS (effective chi-squared statistics); *p*_GATES_, *p* value of GATES (gene-based association test using extended Simes).

### *ARVCF* causally associated with alcohol use

**Figure 1:**
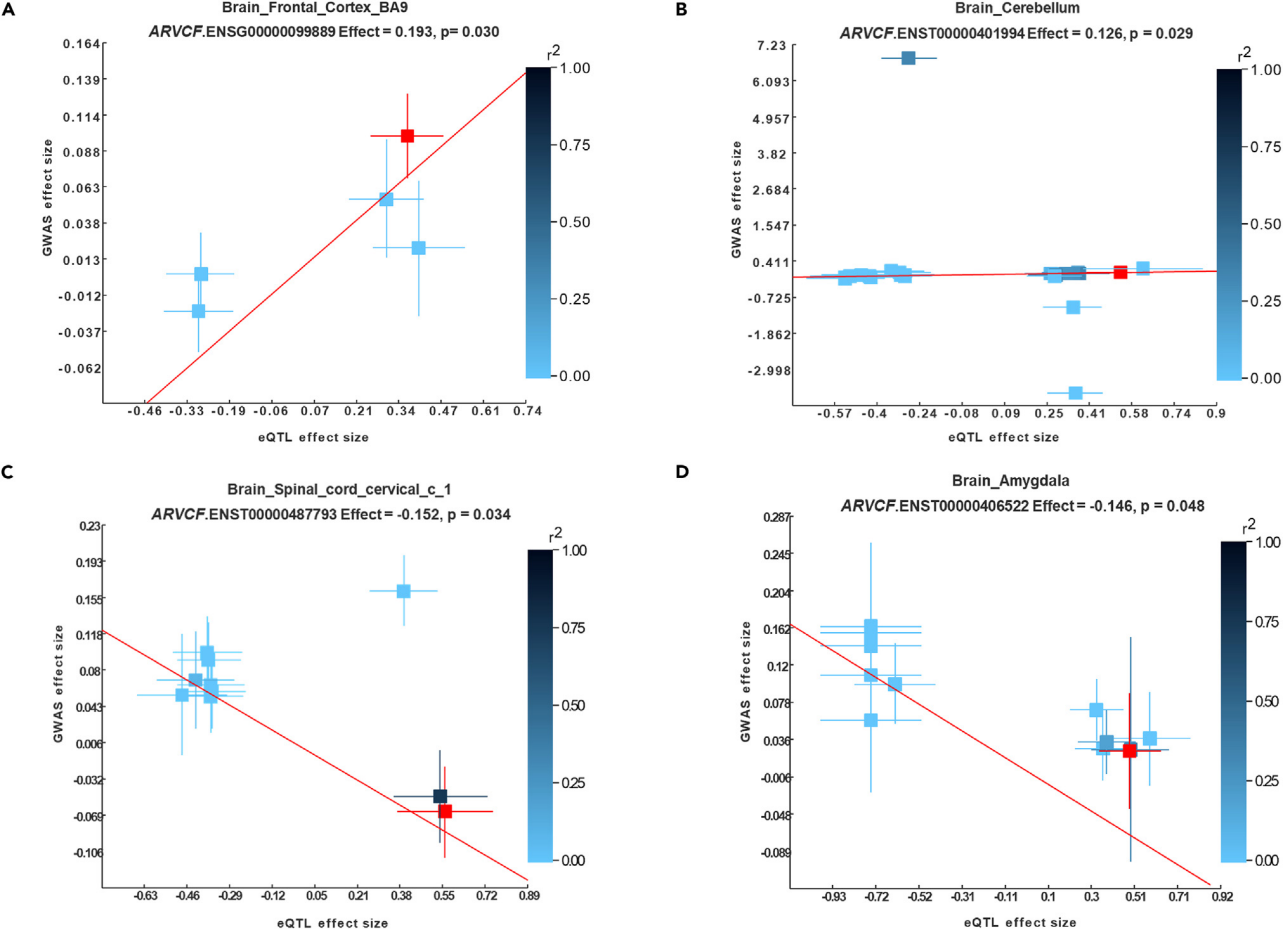
*ARVCF* expression is causally associated with alcohol use in Chinese samples (A) EMIC (effective-median-based Mendelian randomization framework for inferring the causal genes of complex phenotypes) infers the causal effect of *ARVCF* expression in the frontal cortex on alcohol use. (B–D) EMIC analysis infers the causal effect of *ARVCF* transcript expression in cerebellum (B), cervical spinal cord (C), and amygdala (D) on alcohol use. Each rectangle in the plots represents a single-nucleotide polymorphism (SNP). The red rectangle denotes the most significant GWAS variant in the gene. The slope of the red line represents the estimated causal effects determined by EMIC. The varying shades of blue indicate the degree of linkage disequilibrium between the SNP and the most significant variant in association analysis. The error bars depict the standard errors of the coefficient estimates.

Integrative analysis using EMIC, combining GWAS and brain eQTL data from GTEx, suggested a causal relationship between *ARVCF* expression and alcohol use. Our gene-level EMIC analysis revealed a significant causal association of *ARVCF* expression in the frontal cortex with alcohol use (β = 0.193, SE = 0.089, *p* = 0.030) (Figure 1A and Table S5). The transcript-level EMIC analysis also indicated potential causal effects of *ARVCF* transcript expression in the cerebellum (Figure 1B), cervical spinal cord (Figure 1C), and amygdala (Figure 1D) on alcohol use (*p* < 0.05) (Table S6).

### *ARVCF* associated with alcohol dependence in European populations

Replication analysis using data on alcohol dependence from individuals of European ancestry (*N* = 46,568) further supported our findings. Our gene-based association tests showed significant correlations of *ARVCF* expression in the nucleus accumbens (NAc) basal ganglia (*p*_ECS_ = 0.019, *p*_GATES_ = 0.027) and anterior cingulate cortex (*p*_ECS_ = 0.015) with alcohol dependence (Tables 2 and S7). Besides, transcript-based analysis further associated *ARVCF* transcript expression in the cerebellar hemisphere (*p*_GATES_ = 0.010), substantia nigra (*p*_GATES_ = 0.028), and cervical spinal cord (*p*_GATES_ = 0.030) with alcohol dependence (Tables 2 and S8). We also performed EMIC analysis to infer whether *ARVCF* expression in the brain causally associated with alcohol dependence. Our gene-level EMIC analysis indicated a causal relationship between *ARVCF* expression in the NAc and alcohol dependence (β = 0.013, SE = 0.006, *p* = 0.039) (Table 3). Transcript-level EMIC analysis showed that *ARVCF* transcript expression in the amygdala (*p* < 0.05) may also be implicated in alcohol dependence (Table 3). Because most SNPs identified in Chinese population were absent in the European alcohol dependence dataset (Table S9), we primarily focused on gene-level association analysis in the European data. Additionally, we examined the association of *ARVCF* with other related phenotypes using phenome-wide association studies,^21^^,^^22^ which indicated that *ARVCF* is associated with many psychiatric conditions, including smoking and alcohol use (Table S10).

**Table 2:** Results of gene-based and transcript-based *ARVCF* association tests for alcohol dependence in European samples

Brain region	Gene/transcript	Var	*p*_ECS_	*p*_GATES_
**Gene-based association tests**				

Brain_Nucleus_accumbens_basal_ganglia	*ARVCF*	22	0.019	0.027
Brain_Anterior_cingulate_cortex_BA24	*ARVCF*	23	0.015	0.073
Brain_Substantia_nigra	*ARVCF*	13	0.283	0.394
Brain_Substantia_nigra	*ARVCF*	13	0.283	0.394
Brain_Caudate_basal_ganglia	*ARVCF*	57	0.397	0.471
Brain_Cerebellar_Hemisphere	*ARVCF*	14	0.424	0.526
Brain_Amygdala	*ARVCF*	2	0.588	0.638
Brain_Putamen_basal_ganglia	*ARVCF*	21	0.720	0.787
Brain_Hypothalamus	*ARVCF*	48	0.625	0.816
Brain_Spinal_cord_cervical_c_1	*ARVCF*	104	0.911	0.979

**Transcript-based association tests**				

Brain_Cerebellar_Hemisphere	ENST00000473551	16	0.107	0.010
Brain_Substantia_nigra	ENST00000406522	15	0.294	0.028
Brain_Spinal_cord_cervical_c_1	ENST00000495096	105	0.062	0.030
Brain_Nucleus_accumbens_basal_ganglia	ENST00000263207	15	0.087	0.300
Brain_Amygdala	ENST00000495096	9	0.051	0.426
Brain_Putamen_basal_ganglia	ENST00000401994	49	1	1

Var, number of variants within the gene; *p*_ECS_, *p* value of ECS (effective chi-squared statistics); *p*_GATES_, *p* value of GATES (gene-based association test using extended Simes).

**Table 3:** *ARVCF* gene-level and transcript-level EMIC results for alcohol dependence in European samples

Brain region	Gene/transcript	Var	Effect	SE	*p*_EMIC_
**Gene-level EMIC tests**					

Nucleus_accumbens_basal_ganglia	*ARVCF*	22	0.013	0.006	0.039
Brain_Frontal_Cortex_BA9	*ARVCF*	16	−0.004	0.004	0.293

**Transcript-level EMIC tests**					

Brain_Amygdala	ENST00000406522	9	−0.007	0.004	0.049
Brain_Cortex	ENST00000263207	83	−0.007	0.004	0.072
Brain_Cerebellar_Hemisphere	ENST00000263207	84	−0.014	0.010	0.150
Brain_Anterior_cingulate_cortex_BA24	ENST00000473551	116	−0.004	0.004	0.322
Brain_Hippocampus	ENST00000263207	25	−4.015	4.259	0.346
Brain_Caudate_basal_ganglia	ENST00000406522	167	−0.003	0.004	0.494
Brain_Spinal_cord_cervical_c_1	ENST00000487793	105	0.001	0.004	0.838
Brain_Frontal_Cortex_BA9	ENST00000473551	58	−0.002	0.013	0.852
Brain_Cerebellum	ENST00000487793	80	0.000	0.006	0.967
Brain_Nucleus_accumbens_basal_ganglia	ENST00000473551	27	0.001	0.047	0.987
Brain_Putamen_basal_ganglia	ENST00000406522	26	−2.945	436.142	0.995

Var, number of variants within the gene; SE, standard error; *p*_EMIC_, *p* value of EMIC (effective-median-based Mendelian randomization framework for inferring the causal genes of complex phenotypes).

### *Arvcf* is required for alcohol-induced CPP in mice

**Figure 2:**
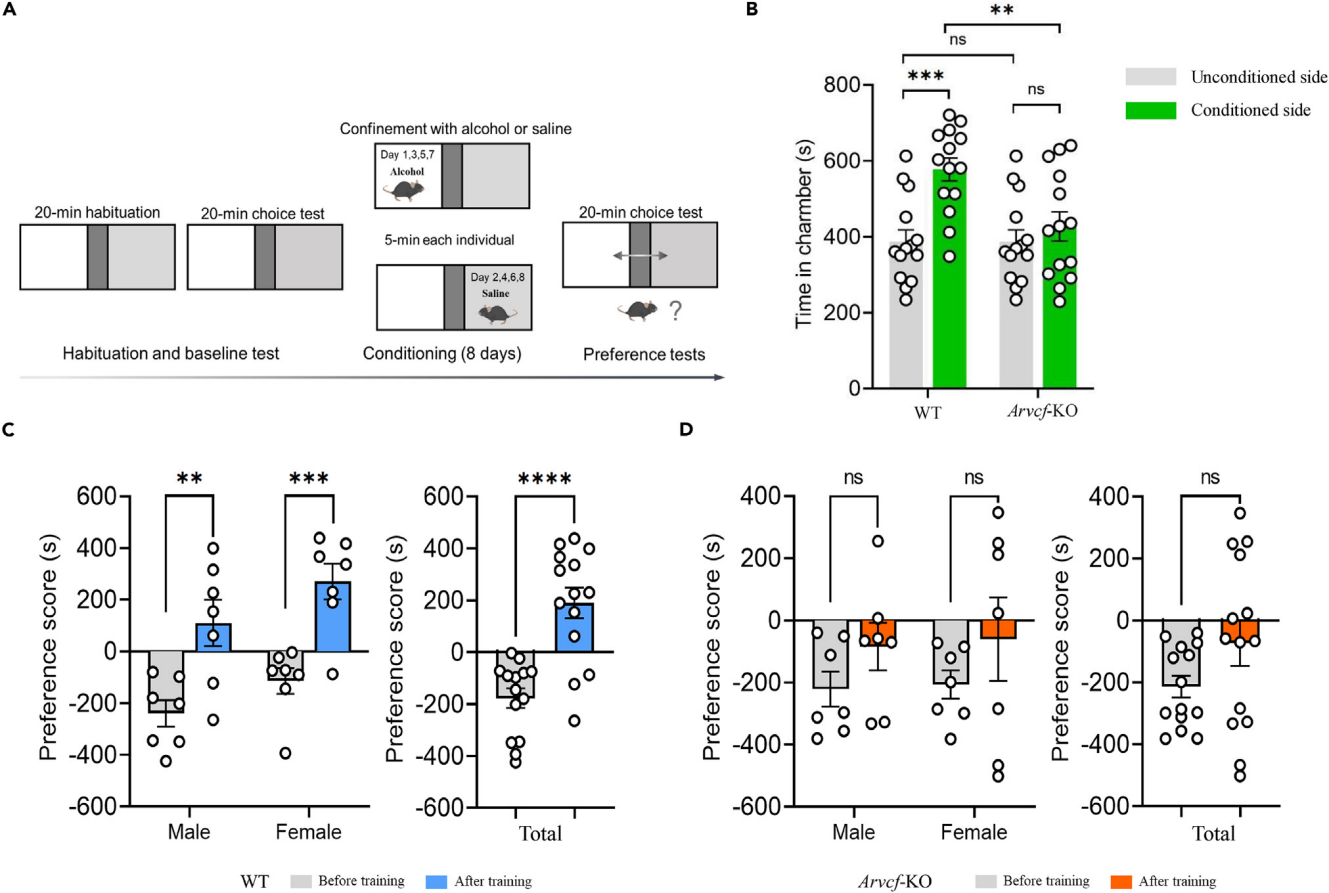
*Arvcf* is required for alcohol-induced conditioned place preference in mice (A) Schematic of ethanol-induced conditioned place preference (CPP). (B) A comparison of time spent in ethanol-conditioned and unconditioned chambers after ethanol-induced CPP training between wild-type (WT, *n* = 14, 7 males and 7 females) and *Arvcf*-KO mice (*n* = 14, 7 males and 7 females). (C and D) Preference scores for the ethanol-paired chamber in wild-type mice (C) (*n* = 14, 7 males and 7 females; left: male and female, respectively; right: male and female pooled) and mice with knockout of *Arvcf* (D) (*n* = 14, 7 males and 7 females; left: male and female, respectively; right: male and female pooled). Data are presented as mean ± SEM; *p* values were determined using the two-tailed unpaired t test. ns, not significant; ∗∗*p* < 0.01, ∗∗∗*p* < 0.001, ∗∗∗∗*p* < 0.0001.

A major driver of alcohol dependence is drug-seeing behavior. To further examine the potential behavioral effect of *Arvcf* in alcohol dependence, we performed an ethanol-induced CPP test. In the CPP paradigm, mice were intraperitoneally injected with either saline or ethanol and placed in distinct spatial chambers distinguished by environmental cues. After conditioning, mice were given unrestricted access to the drug-paired and saline-paired chambers, and CPP was measured by calculating the difference in time spent between the conditioned chamber and the unconditioned chamber (Figure 2A). We also compared the time that mice spent in the unconditioned (exposure to saline) and conditioned (exposure to alcohol) chambers to assess the conditioned reinforcing effect of alcohol.

After conditioning, wild-type (WT) mice showed a significant preference for the chamber that had been paired with alcohol (*p* < 0.001) (Figure 2B). In contrast, *Arvcf*-KO mice displayed no such preference, spending similar amounts of time in the alcohol-paired chamber and the unconditioned side (Figure 2B). Moreover, WT mice spent significantly more time in the conditioned chamber compared to *Arvcf*-KO mice (*p* < 0.01, Figure 2B), with no significant difference in the unconditioned chamber. In addition, a significant CPP score was observed in both male (*p* < 0.01) and female (*p* < 0.001) WT mice, as well as in combined sexes (*p* < 0.0001, Figure 2C), whereas *Arvcf*-KO mice did not exhibit a significant CPP response (Figure 2D). Altogether, these results suggest that *Arvcf* is required for the establishment of alcohol dependence in mice.

## Discussion

In this study, we first revealed that the gene *ARVCF* confers susceptibility to alcohol use in Chinese samples. We further validated the causal relationship between *ARVCF* and alcohol dependence in a large European dataset. More importantly, through the utilization of alcohol-induced CPP tests with CRISPR-Cas9-generated *Arvcf*-KO mice, we demonstrated that *Arvcf* is required for the establishment of alcohol dependence in mice. The central conclusion of this study underscores the essential role of *ARVCF*, a member of the p120ctn protein family, in regulating behaviors related to alcohol dependence.

The p120ctn family of proteins, to which the *ARVCF* belongs, is implicated in the development of dendrites and synapses, both of which play crucial roles in learning, memory, addiction, and neurogenetic disorders.^17^^,^^18^ Building on our prior discovery of a novel nicotine dependence-associated variant in *ARVCF*,^15^ we explored whether *ARVCF* could potentially influence alcohol-related behaviors. In the Chinese population of whole-genome sequencing samples, SNP-based association analysis showed that two variants located in *ARVCF* were significantly associated with drinks per week. One of the variants, rs2531698, is an intronic variant within *ARVCF*, located in 2 kb upstream of the *TANGO2* gene, which is associated with neurodegeneration and intellectual disability.^23^ The other variant, rs116570619, is a synonymous variant. These two variants have not been previously reported in the literature and may serve as potential targets for investigating alcohol use.

Through candidate gene association analysis utilizing Chinese samples, we discovered that *ARVCF* expression in the brain regions known to be affected by alcohol, such as the cortex and hippocampus,^24^ was significantly associated with alcohol use. To further investigate the causal relationship between *ARVCF* expression in the brain and alcohol use, we employed the EMIC method, which is based on the Mendelian randomization framework. Notably, our EMIC analysis consistently reinforced the findings of the association test, indicating a causal relationship between *ARVCF* expression in the frontal cortex and alcohol use.

Our replication analysis, utilizing the dataset on alcohol dependence from individuals of European ancestry, further validated the causal association between *ARVCF* and alcohol use. *ARVCF* was significantly associated with alcohol dependence in both gene-based association test and EMIC analysis. Our findings highlight the significant role of *ARVCF* in alcohol dependence, particularly in its expression within the NAc, a region central to dopamine release and reward-seeking behavior. Alcohol activates the mesocorticolimbic brain reward circuit, which includes dopaminergic projections from the ventral tegmental area in the midbrain to several forebrain structures including the NAc and cortex.^24^^,^^25^ These circuits are involved in the rewarding effects of alcohol. Both our discovery analysis using Chinese samples and our replication analysis using a European dataset consistently indicated that *ARVCF* effects alcohol use through brain regions within these circuits. These genetic data-based analyses collectively establish that *ARVCF* plays an important role in alcohol dependence.

Our animal experiments provide compelling evidence for the important role of *Arvcf* in alcohol dependence, further supporting the findings from human genetic studies. In the CPP test, mice lacking *Arvcf* displayed an absence of alcohol-evoked place preference, and the reinforcing effect of alcohol in *Arvcf*-KO mice was depleted, further confirming the critical role of *ARVCF* in alcohol dependence. These genetic and animal studies together suggested that the effect of *ARVCF* on alcohol dependence is likely attributable to its interaction with the brain’s reward system, particularly the mesocorticolimbic brain reward circuit, which underlies the rewarding effects of alcohol.^24^ Future studies that focus on unraveling the underlying mechanisms of how *ARVCF* controls alcohol dependence could be valuable.

In conclusion, our study identifies *ARVCF* as a susceptibility gene for alcohol dependence. *ARVCF* and its polymorphic sites, such as rs2531698 and rs116570619 identified in this study, may play an important role in alcohol-related disorders and could be valuable for diagnostic and preventative measures of related disorders. Future studies investigating the role of *ARVCF* in the mesocorticolimbic brain reward circuit and its involvement in alcohol dependence may yield further valuable insights. *ARVCF* emerges as a promising drug target for the treatment of alcohol and drug addiction.

### Limitations of the study

This study has several limitations. Firstly, while our dataset is substantial within the context of Chinese alcohol use studies, the sample size is still relatively small compared to European datasets. Future studies with larger Chinese populations and deep sequencing approaches may provide more comprehensive insights. Secondly, the validation analysis in the European population showed less significant results compared to our Chinese cohort. This discrepancy might be attributed to differences in ethnic groups, as allele frequencies and the presence of certain alleles vary greatly between populations. This has also been reflected in our SNP-based analysis, where many SNPs identified in the Chinese population were absent in the European population. Lastly, although we have demonstrated that the *Arvcf* gene plays a crucial role in alcohol dependence in mice, the molecular mechanisms underlying this association were not explored in this study. Future research should aim to elucidate the mechanisms of the *ARVCF* gene in alcohol dependence and other psychiatric conditions.

## Resource availability

### Lead contact

Further information and request for resources should be addressed to Prof. Ming D. Li at ml2km@zju.edu.cn.

### Materials availability

The study did not generate any new materials.

### Data and code availability

• Datasets used in this study are listed in the STAR methods section and supplementary files.
• This paper does not report original code.
• Any additional information required to reanalyze the data reported in this paper or reproduce the results is available from the lead contact upon request.

## Acknowledgments

This study was supported in part by the 10.13039/501100001809National Natural Science Foundation of China (82271560), China Precision Medicine Initiative (2016YFC0906300), Research Center for Air Pollution and Health of Zhejiang University, and the State Key Laboratory for Diagnosis and Treatment of Infectious Diseases of the First Affiliated Hospital of Zhejiang University.

## Author contributions

X.S. and Y.W. conducted the experiments; X.S. and Z.Y. participated in data analysis; Z.Y., W.Y., and M.D.L. provided the resources and managed the project; X.S. and M.D.L. participated in article writing and editing; M.D.L. conceived the study and was involved in every step of the study. All authors approved the article as submitted.

## Declaration of interests

The authors declare no competing interests.

## STAR★Methods

### Key resources table

**Table:** 

REAGENT or RESOURCE	SOURCE	IDENTIFIER
**Deposited data**		

GTEx v8 eQTL data	KGGSEE website	https://pmglab.top/kggsee/#/download
European alcohol dependence GWAS summary statistics	Psychiatric Genomics Consortium	https://pgc.unc.edu/for-researchers/download-results/

**Experimental models: Organisms/strains**		

Mouse: C57BL/6	GemPharmatech	N/A
Mouse: *Arvcf* knockout	GemPharmatech	N/A

**Software and algorithms**		

PLINK v1.9	Chang et al.^26^	https://www.cog-genomics.org/plink/
ECS on KGGSEE v1.0	Li et al.^27^	https://pmglab.top/kggsee/#/
GATES on KGGSEE v1.0	Li et al.^28^	https://pmglab.top/kggsee/#/
EMIC on KGGSEE v1.0	Lin et al.^29^	https://pmglab.top/kggsee/#/
R	N/A	https://www.r-project.org
LDBlockShow	Dong et al.^30^	https://github.com/BGI-shenzhen/LDBlockShow
GraphPad Prism 9.0.0	GraphPad Software lnc	https://www.graphpad.com/
ANY-maze 6.0	Stoelting	https://stoeltingco.com/Neuroscience/ANY-maze

### Experimental model and study participant details

#### Subjects

DNA samples and phenotypic data were collected from Chinese participants. All participants were recruited from Shanxi Province between 2012 and 2014, as detailed in our previous study.^15^^,^^31^ The alcohol-related questionnaires, including drinking status and drinks per week, were administered, and demographic characteristics such as age and gender were obtained from each individual. Participants diagnosed with psychiatric disorders as defined by the DSM-IV, such as schizophrenia and major depressive disorders, were excluded from the study. Only males were included in the study due to few females drinking either in our study or in China.^32^ In total, 1,329 biologically unrelated subjects were included for whole genome-sequencing (WGS).

#### Animals

The Wild-type (WT, C57BL/6) mice were purchased from GemPharmatech (Nanjing, China). The *Arvcf*-KO mice were generated using the CRISPR/Cas9 system and provided by GemPharmatech. All mice were socially housed (3–5 animals per cage) and maintained on a 12-h light-dark cycle (light on from 7:00 to 19:00) with food and water provided *ad libitum*. The project was approved by the Animal Care and Use Committee of the First Affiliated Hospital of Zhejiang University (Approval #: 2023-596).

### Method details

#### Alcohol phenotypes

Currently consuming any alcoholic beverages was defined as being a drinker, and drinks per week was defined as the average number of drinks a participant reported drinking each week, including all types of alcohol. While a range of response for drinks per week was recorded, the midpoint was used (e.g., 2 drinks per week on average instead of a range of 1–3 drinks per week). This phenotype was left-anchored at 1 and log-transformed before analysis, as previously described.^33^

#### Genetic data and variants association analysis

Whole genome sequencing and quality control were performed as described in our recent report.^34^ Briefly, genomic DNA was extracted from blood and sequenced on the Illumina HiSeqX platform. After quality control, all sequencing reads were aligned to the NCBI build 37 of the human reference genome using BMA, and variants were determined using GATK, as detailed in our recent report.^34^ The SNP-based association analysis was performed using PLINK v1.9^26^ under an additive genetic model. SNPs with a minor allele frequency (MAF) < 0.01, SNPs and samples with a call rate <0.99, and P-value for Hardy-Weinberg equilibrium (HWE) < 5 × 10^−6^ were excluded from the analysis.^15^^,^^35^ After implementing quality control procedures, we analyzed data from 470 nondrinkers and 834 alcohol drinkers, along with their corresponding drinks per week, while accounting for age and the first three multidimensional scaling (MDS) components as covariates.^15^ We extracted all variants located in *ARVCF* gene region (including ±2 kb flanking regions) from the whole genome-sequencing analysis data for further analyses. Regional association plot was generated using LDBlockShow.^30^

#### Gene-based association test

For our Chinese dataset, the SNP-based association analysis results in the *ARVCF* gene region were applied to gene-based and transcript-based association testing using KGGSEE software, which employs the approaches of a rapid and powerful Gene-based Association Test using Extended Simes procedure (GATES)^28^ and Effective Chi-square Statistics (ESC).^27^ The gene- and transcript-based eQTL summary statistics from GTEx v8 brain tissues were utilized. SNPs with MAF ≤0.01 and eQTL summary statistics with P-value >0.05 were excluded from the analysis. The dataset used for validation on alcohol dependence was the largest so far publicly available genome-wide association study on DSM-IV-diagnosed alcohol dependence, including 14,904 individuals with alcohol dependence and 37,944 controls from 28 case-control and family-based studies.^36^ We applied the same procedures as those used for the Chinese data to generate gene-based association estimates for *ARVCF* in relation to alcohol dependence.

#### Effective-median-based mendelian randomization analysis

We used the EMIC (Effective-median-based Mendelian randomization framework for Inferring the Causal genes of complex phenotypes) to infer the causal effect of *ARVCF* gene expression on alcohol use.^29^ To achieve this, the pre-calculated *cis*-eQTLs (*p* ≤ 0.05) in 13 brain tissues with gene-level and transcript-level expression from GTEx (V8) and SNPs (MAF >0.01) within the *ARVCF* gene were utilized for the EMIC analysis. In the EMIC analysis, variants affecting *ARVCF* expression, as well as their effect sizes and standard errors for alcohol-related phenotypes, were used as input. This allowed EMIC to estimate the causal effect of gene expression or transcript levels on alcohol use. Since allele frequencies for alcohol dependence were not reported, we utilized the allele frequencies from the European 1000 Genomes dataset.^37^ We applied both the Chinese data and dataset of alcohol dependence from European ancestry^36^ to EMIC analysis.

#### Mouse experiments

Behavioral assays were conducted when the animals were 8 weeks old. The mice were placed in the testing room for habituation at least 30 min before the test and were handled once per day for at least 4 days before the behavioral test. The behavioral tasks were performed during the light phase. In the ethanol-induced CPP test, both adult *Arvcf*-KO and WT male and female mice were used. Ethanol was administered via intraperitoneal injection at a dose of 2 g/kg (20% solution in saline).^38^ We used a three-chamber CPP apparatus consisting of two large boxes that act as conditioning chambers separated by a small middle chamber serving as a transit route between boxes. The two side chambers (450 × 450 × 450 mm) had different wall colors (white and black) and different floor textures, with one having a grid mental floor and the other having a floor with horizontal metal strips. The paradigm consisted of four phases: habituation, pre-conditioning, conditioning, and post-conditioning. During the phases of habituation and pre-conditioning (day 1–2), guillotine doors were removed, and the animal was placed in the middle chamber to allow it to freely explore the entire apparatus for 20 min. Animal behavior was record by a video-tracking system (ANY-maze 6.0), and the time spent in each chamber was automatically calculated on day 2 as the baseline for the CPP test. Mice were paired with ethanol on the least preferred side, while the other side was the saline-paired chamber. During the conditioning phase (day 3–10), mice received alternating intraperitoneal injections of saline or ethanol and were immediately confined to the saline- or drug-paired chamber for 5 min. In the post-conditioning phase (day 11), the procedure was the same as in the pre-conditioning phase, i.e., mice were allowed access to both chamber for 20 min. Preference scores were calculated by subtracting the time spent in the saline-paired side from the ethanol-paired side.^38^

### Quantification and statistical analysis

PLINK v1.9 was used for SNP-based association analysis.^26^ The KGGSEE software was employed for gene-based and transcript-based association tests,^27^^,^^28^ as well as for EMIC analyses.^29^ In the animal studies, data are presented as Mean ± Standard Error of the Mean (SEM), as indicated in the figure legends. Two-tailed unpaired *t*-test were used for two-group comparisons.
