# Supplementary material for: Identification and validation of a novel gene ARVCF associated with alcohol dependence among Chinese population
Source: iScience. 2024 Sep 16;27(10):110976. doi: 10.1016/j.isci.2024.110976 (PMC11490727; doi:10.1016/j.isci.2024.110976)
Supplement: Document S1. Figures S1 and Tables S1–S10 [file mmc1.pdf]

iScience, Volume *27*

Supplemental information

Identification and validation of a novel gene *ARVCF* associated with alcohol dependence among Chinese population

Xiaoqiang Shi, Yan Wang, Zhongli Yang, Wenji Yuan, and Ming D. Li

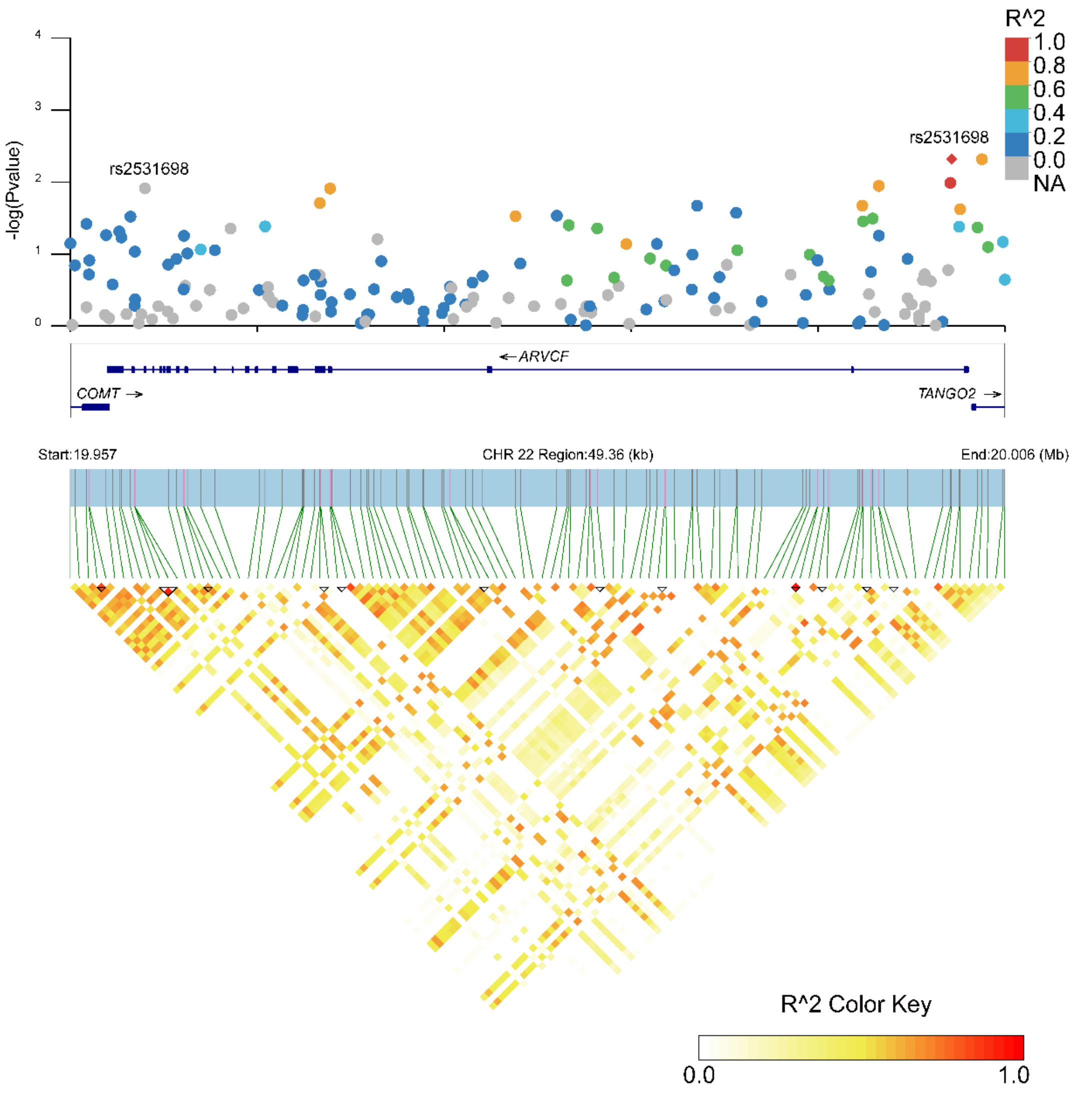

Figure S1. Regional association and linkage disequilibrium plot in the *ARVCF* locus with drinks per week in the Chinese population, related to STAR Methods.
Association of SNPs in the *ARVCF* region (including ±2 kb flanking regions) with drinks per week in Chinese individuals was illustrated. The two significant SNPs are indicated in the figure (top). The top SNP (rs2531698) is highlighted in rhombus, and the other SNPs are colored according to their degree of linkage disequilibrium (r^2) with the top SNP. In the below linkage disequilibrium (LD) plot, LD block is marked by a black triangle.

Table S1. Characteristics of Chinese samples used in the study, related to STAR Methods.

Characteristic	Overall<br>N = 1,329	NA<br>N = 25	Control<br>N = 470	Alcohol drinking<br>N = 834
**Age**	41 ± 8	42 ± 7	42 ± 8	40 ± 8
**Drinks per week**				
-	496	25	470	1
< 1	134	-	-	134
1-3	209	-	-	209
4-7	221	-	-	221
8-10	89	-	-	89
11-14	61	-	-	61
15-17	16	-	-	16
18-21	25	-	-	25
> 21	78	-	-	78

NA = not available

Table S2. SNP association analysis results in the *ARVCF* region with drinks per week and alcohol drink status in Chinese individuals, related to STAR Methods.

Phenotype	SNP	CHR	BP	A1	A2	MAF	BETA	SE	P
Drinks per week	rs2531698	22	20003334	A	G	0.355	0.107	0.038	4.79E-03
Drinks per week	rs116570619	22	19960722	A	G	0.015	-0.409	0.163	0.012
Drinks per week	rs75686659	22	19973009	T	C	0.013	-0.313	0.167	0.062
Drinks per week	Var-22-20001904	22	20001904	A	G	0.030	0.157	0.120	0.191
Drinks per week	Var-22-20001613	22	20001613	A	G	0.015	0.056	0.152	0.715
Alcohol drink	rs117359238	22	19965295	A	G	0.018	-0.436	0.292	0.136
Alcohol drink	Var-22-20001616	22	20001616	G	C	0.016	0.498	0.349	0.153
Alcohol drink	Var-22-20001904	22	20001904	A	G	0.030	-0.275	0.235	0.242
Alcohol drink	rs369057137	22	20002494	A	G	0.016	0.245	0.322	0.446
Alcohol drink	rs143369598	22	20001219	C	G	0.014	-0.226	0.313	0.471

SNP association analysis including a ±2 kb flanking region of the *ARVCF* gene (clumped for linkage disequilibrium ($r2 < 0.01$, within 250kb genomic window)). CHR = chromosome; BP = base pair; A1 = minor allele; A2 = reference allele; MAF = minor allele frequency; SE = standard error; P = p-value.

Table S3. Results of gene-based *ARVCF* association tests in Chinese samples, related to Table 1

Phenotype	Brain region	Var	ECSP	GATESP
Drinks per week	**Brain_Spinal_cord_cervical_c_1**	45	**0.013**	**2.93E-04**
Alcohol drink	**Brain_Frontal_Cortex_BA9**	5	**0.012**	**0.004**
Alcohol drink	**Brain_Cortex**	31	0.082	**0.007**
Alcohol drink	**Brain_Cerebellum**	40	0.105	**0.029**
Alcohol drink	Brain_Hypothalamus	36	0.359	0.074
Alcohol drink	Brain_Hippocampus	15	0.336	0.140
Drinks per week	Brain_Caudate_basal_ganglia	38	0.321	0.152
Alcohol drink	Brain_Substantia_nigra	10	0.313	0.198
Alcohol drink	Brain_Nucleus_accumbens_basal_ganglia	8	0.295	0.344
Drinks per week	Brain_Frontal_Cortex_BA9	5	0.306	0.369
Drinks per week	Brain_Hypothalamus	36	0.194	0.407
Drinks per week	Brain_Amygdala	2	0.389	0.433
Alcohol drink	Brain_Spinal_cord_cervical_c_1	45	0.417	0.446
Drinks per week	Brain_Cerebellum	40	0.619	0.450
Drinks per week	Brain_Substantia_nigra	10	0.617	0.575
Drinks per week	Brain_Nucleus_accumbens_basal_ganglia	8	0.564	0.593
Drinks per week	Brain_Anterior_cingulate_cortex_BA24	20	0.644	0.648
Alcohol drink	Brain_Caudate_basal_ganglia	38	0.571	0.663
Alcohol drink	Brain_Amygdala	2	0.834	0.681
Alcohol drink	Brain_Putamen_basal_ganglia	6	0.577	0.746
Alcohol drink	Brain_Cerebellar_Hemisphere	16	0.631	0.755
Alcohol drink	Brain_Anterior_cingulate_cortex_BA24	20	0.682	0.803
Drinks per week	Brain_Cerebellar_Hemisphere	16	0.757	0.835
Drinks per week	Brain_Cortex	31	0.967	0.911
Drinks per week	Brain_Hippocampus	15	0.890	0.970
Drinks per week	Brain_Putamen_basal_ganglia	6	0.846	0.984

Var = number of variants within the gene; ECSP = p-value of ECS (Effective Chi-square Statistics); GATESP = p-value of GATES (Gene-based Association Test using Extended Simes).

Table S4. Results of transcript-based *ARVCF* association tests in Chinese samples, related to Table 1.

Phenotype	Brain region	Ensembl transcript ID	Var	ECSP	GATESP
Drinks per week	**Brain_Hippocampus**	ENST00000406522	13	**0.022**	**4.07E-04**
Drinks per week	**Brain_Spinal_cord_cervical_c_1**	ENST00000487793	25	**0.002**	**6.40E-04**
Alcohol drink	**Brain_Cortex**	ENST00000473551	8	0.055	**0.022**
Alcohol drink	**Brain_Frontal_Cortex_BA9**	ENST00000495096	41	0.054	**0.044**
Alcohol drink	Brain_Cerebellum	ENST00000401994	53	0.095	0.105
Alcohol drink	Brain_Caudate_basal_ganglia	ENST00000401994	7	0.249	0.271
Drinks per week	Brain_Hypothalamus	ENST00000263207	9	0.438	0.291
Alcohol drink	Brain_Hypothalamus	ENST00000473551	30	1.000	0.496
Drinks per week	Brain_Nucleus_accumbens_basal_ganglia	ENST00000401994	30	1.000	0.692
Alcohol drink	Brain_Cerebellar_Hemisphere	ENST00000462319	41	1.000	0.733
Drinks per week	Brain_Putamen_basal_ganglia	ENST00000401994	45	0.117	0.736
Alcohol drink	Brain_Hippocampus	ENST00000406522	13	1.000	0.920
Alcohol drink	Brain_Amygdala	ENST00000263207	8	1.000	1.000
Alcohol drink	Brain_Anterior_cingulate_cortex_BA24	ENST00000487793	5	1.000	1.000
Alcohol drink	Brain_Nucleus_accumbens_basal_ganglia	ENST00000406522	30	0.911	1.000
Alcohol drink	Brain_Putamen_basal_ganglia	ENST00000495096	7	1.000	1.000
Alcohol drink	Brain_Spinal_cord_cervical_c_1	ENST00000487793	25	1.000	1.000
Alcohol drink	Brain_Substantia_nigra	ENST00000495096	12	1.000	1.000
Drinks per week	Brain_Substantia_nigra	ENST00000401994	24	1.000	1.000

Var = number of variants within the gene; ECSP = p-value of ECS (Effective Chi-square Statistics); GATESP = p-value of GATES (Gene-based Association Test using Extended Simes).

Table S5. *ARVCF* gene-level EMIC results in Chinese samples, related to Figure 1.

Phenotype	Brain region	Var	Effect	SE	P_EMIC
Alcohol drink	**Brain_Frontal_Cortex_BA9**	5	0.193	0.089	**0.030**
Alcohol drink	Brain_Hippocampus	15	-0.088	0.051	0.083
Alcohol drink	Brain_Caudate_basal_ganglia	38	-0.068	0.047	0.146
Drinks per week	Brain_Spinal_cord_cervical_c_1	45	-0.083	0.061	0.174
Drinks per week	Brain_Anterior_cingulate_cortex_BA24	20	-0.048	0.039	0.215
Drinks per week	Brain_Cerebellum	40	-0.102	0.089	0.251
Alcohol drink	Brain_Cortex	31	0.057	0.051	0.262
Drinks per week	Brain_Nucleus_accumbens_basal_ganglia	8	-0.078	0.080	0.324
Alcohol drink	Brain_Hypothalamus	36	0.066	0.069	0.339
Alcohol drink	Brain_Nucleus_accumbens_basal_ganglia	8	-0.051	0.068	0.449
Alcohol drink	Brain_Spinal_cord_cervical_c_1	45	-0.031	0.049	0.524
Drinks per week	Brain_Hippocampus	15	-0.033	0.055	0.547
Drinks per week	Brain_Caudate_basal_ganglia	38	-0.032	0.058	0.572
Drinks per week	Brain_Cerebellar_Hemisphere	16	0.034	0.070	0.628
Alcohol drink	Brain_Cerebellum	40	-0.101	0.233	0.665
Drinks per week	Brain_Hypothalamus	36	0.031	0.074	0.671
Drinks per week	Brain_Frontal_Cortex_BA9	5	-0.018	0.083	0.826
Alcohol drink	Brain_Cerebellar_Hemisphere	16	0.009	0.054	0.869
Alcohol drink	Brain_Anterior_cingulate_cortex_BA24	20	-0.006	0.035	0.872
Alcohol drink	Brain_Substantia_nigra	10	0.053	0.337	0.875
Alcohol drink	Brain_Putamen_basal_ganglia	6	-0.016	0.105	0.876
Drinks per week	Brain_Cortex	31	0.008	0.057	0.887
Drinks per week	Brain_Putamen_basal_ganglia	6	-0.015	0.137	0.915
Drinks per week	Brain_Substantia_nigra	10	-0.025	0.684	0.971

Var = number of variants within the gene; SE = standard error; P_EMIC = p-value of EMIC (Effective-median-based Mendelian randomization framework for Inferring the Causal genes of complex phenotypes).

Table S6. *ARVCF* transcript-level EMIC results in Chinese samples, related to Figure 1.

Phenotype	Brain region	Ensembl transcript ID	Var	Effect	SE	P_EMIC
Alcohol drink	**Brain_Cerebellum**	ENST00000401994	70	0.126	0.058	**0.029**
Drinks per week	**Brain_Spinal_cord_cervical_c_1**	ENST00000487793	47	-0.152	0.072	**0.034**
Drinks per week	**Brain_Amygdala**	ENST00000406522	7	-0.146	0.074	**0.048**
Alcohol drink	Brain_Cerebellar_Hemisphere	ENST00000263207	72	0.090	0.052	0.080
Alcohol drink	Brain_Amygdala	ENST00000487793	7	-1.157	0.676	0.087
Drinks per week	Brain_Putamen_basal_ganglia	ENST00000406522	7	-0.172	0.102	0.094
Alcohol drink	Brain_Hippocampus	ENST00000263207	7	-0.074	0.044	0.097
Drinks per week	Brain_Caudate_basal_ganglia	ENST00000263207	99	0.106	0.065	0.104
Drinks per week	Brain_Nucleus_accumbens_basal_ganglia	ENST00000406522	12	-0.107	0.066	0.105
Drinks per week	Brain_Substantia_nigra	ENST00000401994	12	0.108	0.068	0.109
Alcohol drink	Brain_Caudate_basal_ganglia	ENST00000263207	99	-0.087	0.055	0.116
Alcohol drink	Brain_Hypothalamus	ENST00000473551	37	0.112	0.071	0.117
Drinks per week	Brain_Hypothalamus	ENST00000401994	37	0.093	0.060	0.122
Drinks per week	Brain_Cerebellar_Hemisphere	ENST00000487793	72	-0.198	0.129	0.125
Alcohol drink	Brain_Nucleus_accumbens_basal_ganglia	ENST00000406522	12	-0.089	0.061	0.144
Drinks per week	Brain_Cortex	ENST00000495096	62	-0.094	0.071	0.188
Alcohol drink	Brain_Cortex	ENST00000473551	62	0.127	0.097	0.193
Drinks per week	Brain_Anterior_cingulate_cortex_BA24	ENST00000473551	92	-0.053	0.043	0.225
Drinks per week	Brain_Cerebellum	ENST00000462319	70	-0.061	0.055	0.267
Alcohol drink	Brain_Anterior_cingulate_cortex_BA24	ENST00000406522	92	-0.055	0.050	0.272
Drinks per week	Brain_Hippocampus	ENST00000406522	7	-0.094	0.099	0.343
Alcohol drink	Brain_Substantia_nigra	ENST00000487793	12	-0.059	0.065	0.359
Drinks per week	Brain_Frontal_Cortex_BA9	ENST00000401994	41	0.092	0.101	0.366
Alcohol drink	Brain_Putamen_basal_ganglia	ENST00000406522	7	-0.055	0.075	0.459
Alcohol drink	Brain_Spinal_cord_cervical_c_1	ENST00000495096	47	-0.030	0.050	0.550
Alcohol drink	Brain_Frontal_Cortex_BA9	ENST00000495096	41	-0.028	0.052	0.598

Var = number of variants within the gene; SE = standard error; P_EMIC = p-value of EMIC (Effective-median-based Mendelian randomization framework for Inferring the Causal genes of complex phenotypes), this is the minimum p-value among all transcripts of the gene.

Table S7. Results of gene-based *ARVCF* association tests for alcohol dependence in European samples, related to Table 2.

Brain region	Var	ECSP	GATESP
**Brain_Nucleus_accumbens_basal_ganglia**	22	**0.019**	**0.027**
**Brain_Anterior_cingulate_cortex_BA24**	23	**0.015**	0.073
Brain_Substantia_nigra	13	0.283	0.394
Brain_Substantia_nigra	13	0.283	0.394
Brain_Caudate_basal_ganglia	57	0.397	0.471
Brain_Cerebellar_Hemisphere	14	0.424	0.526
Brain_Amygdala	2	0.588	0.638
Brain_Putamen_basal_ganglia	21	0.720	0.787
Brain_Hypothalamus	48	0.625	0.816
Brain_Spinal_cord_cervical_c_1	104	0.911	0.979

Var = number of variants within the gene; ECSP = p-value of ECS (Effective Chi-square Statistics); GATESP = p-value of GATES (Gene-based Association Test using Extended Simes).

Table S8. Results of transcript-based *ARVCF* association tests for alcohol dependence in European samples, related to Table 2.

Brain region	Ensembl transcript ID	Var	ECSP	GATESP
**Brain_Cerebellar_Hemisphere**	ENST00000473551	16	0.107	**0.010**
**Brain_Substantia_nigra**	ENST00000406522	15	0.294	**0.028**
**Brain_Spinal_cord_cervical_c_1**	ENST00000495096	105	0.062	**0.030**
Brain_Nucleus_accumbens_basal_ganglia	ENST00000263207	15	0.087	0.300
Brain_Amygdala	ENST00000495096	9	0.051	0.426
Brain_Putamen_basal_ganglia	ENST00000401994	49	1.000	1.000

Var = number of variants within the gene; ECSP = p-value of ECS (Effective Chi-square Statistics); GATESP = p-value of GATES (Gene-based Association Test using Extended Simes).

Table S9. Information on SNPs in the *ARVCF* region identified in the Chinese population present in the European dataset, related to Table S2 and STAR Methods.

SNP	CHR	BP	P
rs2531698	22	20003334	0.406
rs116570619	22	19960722	NA
rs75686659	22	19973009	NA
Var-22-20001904	22	20001904	NA
Var-22-20001613	22	20001613	NA
rs117359238	22	19965295	NA
Var-22-20001616	22	20001616	NA
Var-22-20001904	22	20001904	NA
rs369057137	22	20002494	NA
rs143369598	22	20001219	NA

CHR = chromosome; BP = base pair; P = p-value from the European alcohol dependence dataset. NA = not available.

Table S10. PheWAS table of the *ARVCF* gene in the psychiatric domain, related to Table 2.

PMID	Year	Domain	Subject	Trait	P-value	N
30643256	2019	Psychiatric	**Depression**	Depressive symptoms (univariate)	5.57E-08	1067913
30643256	2019	Psychiatric		Depressive symptoms (MA GWAMA)	7.85E-07	1067913
29662059	2018	Psychiatric		Broad depression	0.0001098	322580
30643256	2019	Psychiatric	**Well-being**	Well-being spectrum	8.88E-07	2311184
31427789	2019	Psychiatric		Happiness and subjective well-being - General happiness with own health	0.0012917	126477
31427789	2019	Psychiatric		Happiness and subjective well-being - General happiness	0.046153	126132
30643256	2019	Psychiatric	**Neuroticism**	Neuroticism (univariate)	3.442E-05	523783
27089181	2016	Psychiatric		Neuroticism	5.593E-05	170911
30643256	2019	Psychiatric		Neuroticism (MA GWAMA)	0.0017104	523783
30867560	2019	Psychiatric	**Anxiety/Worry/Nervous**	Anxiety/tension factors	0.0002638	270059
30867560	2019	Psychiatric		Worry/vulnerability factors	0.0002638	270059
29500382	2018	Psychiatric		Nervous feelings (NERV-FEEL)	0.0014987	264858
31427789	2019	Psychiatric	**Smoking**	Likelihood of resuming smoking	0.003668	93128
31427789	2019	Psychiatric		Ever smoked	0.0082273	385013
31427789	2019	Psychiatric		Past tobacco smoking	0.015252	355594
31427789	2019	Psychiatric	**Alcohol**	Reason for reducing amount of alcohol drunk: Other reason	0.018885	142645
30482948	2018	Psychiatric		Alcohol dependency (fixed effect model for unrelated genotyped individuals)	0.023464	34556
30482948	2018	Psychiatric		Alcohol dependency (full discovery samples)	0.029181	52848
30952852	2019	Psychiatric	**Sleep**	Sleep efficiency	0.0083553	84810
27992416	2017	Psychiatric		Sleep duration	0.016661	111975

30804565	2019	Psychiatric		Daytime napping	0.018151	386577
29970889	2018	Psychiatric	**Loneliness**	Loneliness (MTAG)	0.0093245	487647
29970889	2018	Psychiatric		Loneliness	0.030054	445024
31427789	2019	Psychiatric	**Others**	Ever unenthusiastic/disinterested for a whole week	0.0030722	123848
31427789	2019	Psychiatric		Traumatic events - Felt loved as child	0.011548	126348
31427789	2019	Psychiatric		Morning/evening person (chronotype)	0.014282	345148
30804565	2019	Psychiatric		Morningness	0.023441	345552
31427789	2019	Psychiatric		Getting up in morning	0.027295	385494
31427789	2019	Psychiatric		Risk taking	0.0288	372651
30696823	2019	Psychiatric		Chronotype	0.035228	449732
30804565	2019	Psychiatric		Ease of getting up in the morning	0.043262	385949
29500382	2018	Psychiatric		Guilty feelings (GUILT)	0.047517	265139
20732625	2010	Psychiatric		Attention deficit hyperactivity disorder	0.048564	5415

Display up to three items if a subject has more than three items. Sample size (N) exceeds 1000.
